# Divergent nucleic acid allocation in juvenile insects of different metamorphosis modes

**DOI:** 10.1038/s41598-021-89736-w

**Published:** 2021-05-13

**Authors:** Manuel Villar-Argaiz, Manuel J. López-Rodríguez, J. Manuel Tierno de Figueroa

**Affiliations:** 1 Departamento de Ecología, Facultad de Ciencias, Universidad de Granada, 18071 Granada, Spain; 2 Departamento de Zoología, Facultad de Ciencias, Universidad de Granada, 18071 Granada, Spain

**Keywords:** Ecosystem ecology, Evolutionary ecology

## Abstract

Nucleic acids help clarify variation in species richness of insects having different metamorphosis modes, a biological conundrum. Here we analyse nucleic acid contents of 639 specimens of aquatic insects collected from four high mountain streams of Sierra Nevada in southern Spain to test whether the allocation to RNA or DNA content differs during ontogeny between juvenile insects undergoing direct (hemimetabolous) or indirect (holometabolous) metamorphosis. The results show that RNA content as a function of body mass was negatively correlated to insect body length in four out of six and three out of six of the holometabolan and hemimetabolan taxa, respectively. Although no significant differences in RNA content were found between holometabolans and hemimetabolans, the significant interaction between body length and metamorphosis mode for RNA and RNA:DNA indicates a strong ontogenetic component to RNA allocation. In addition, our finding of lower DNA content in holometabolans relative to hemimetabolans agree with the analysis of empirical genome data in aquatic and terrestrial insects, and extend to this class of arthropods the “growth rate-genome size-nutrient limitation” hypothesis that differences in allocation between RNA and DNA may reflect fundamental evolutionary trade-off of life-history strategies associated with high growth rates (and RNA content) in holometabolans at the expense of diminished genome sizes.

## Introduction

In pterygote insects juveniles undergo greater or lesser morphological differences with respect to adult forms by undergoing one of two modes of metamorphosis: either indirect metamorphosis (holometaboly) or direct (hemimetaboly). Of all known living insects, 80.7% are holometabolans, 19.2% are hemimetabolans, and 0.1% are non-pterygote ametabolans Archaeognatha and Zygentoma^1^. This radical transformation from juvenile to adult has long been studied from the standpoint of morphology, physiology, and regulation of development, while recent studies have made progress elucidating the molecular and hormonal mechanisms driving moulting and metamorphosis^2,3^. However, many questions have been neglected regarding how the mode of metamorphosis relates to the biochemical composition and in turn affects key life-history traits and ecological fitness.

At the core of Biology as a science lies the idea that each organism’s life-history strategies require biochemical changes and shifts in elemental components^4,5^. In the field of biological stoichiometry, the growth-rate hypothesis (GRH) states that organisms with high specific growth rates demand large amounts of phosphorus (P) to build the RNA needed to sustain rapid protein synthesis and growth^4,6^. The GRH holds for many invertebrates, helping to explain biochemical differences among and within species^7,8^. Recent work has demonstrated differences more pronounced than previously thought regarding the development of holometabolans in comparison to hemimetabolans in terms of P content variability, and presumably in RNA content^9^. Because holometabolous insects grow almost twice as much in length at each moult as hemimetabolous do^10^, we might expect the former to have considerably more RNA.

The relation between RNA and DNA content has been used widely as a potential indicator of growth in numerous organisms^11–13^. However, profound causal links between RNA and DNA content have been cited by one research team as an explanation, among others^14^ (the “bulk DNA” or the “selfish DNA”), for the variation in the genome size of organisms^15^. These latter researchers hypothesised that the evolutionary mechanism by which intracellular allocation of P from DNA to RNA would accelerate growth rates but reduce the genome size in fast-growing cladocerans compared to long-lived copepods facing chronic P limitation (“growth rate-genome size-nutrient limitation” hypothesis). In our work, we extend this hypothesis by predicting that large differences in nucleic acid (NA) investment and allocation should accompany the ontogenetic development of insects having different modes of metamorphosis. Specifically, we expect to find an overall lower DNA content in holometabolous compared to hemimetabolous taxa. In addition, we predict significant body length × metamorphosis mode effects on RNA and RNA:DNA ratio as an indication of a varying RNA investment over the ontogeny of holo vs. hemimetabolans. To test these predictions, we compared the NA content of 639 specimens of aquatic nymphs or larvae belonging to six holometabolan and six hemimetabolan taxa in four basins of Sierra Nevada National Park in Spain (Table 1). We further tested our prediction of lower genome sizes (C-value, i.e. quantity of nuclear haploid DNA) in holometabolans vs. hemimetabolans using a genome database by Gregory^16^ containing a total of 1335 insect genetic analyses (Animal Genome Size Database, www.genomesize.com).

**Table 1:** Aquatic insects used in the nucleic acid analyses in this study.

Taxa	Order	Metamorphosis mode	Body mass (mg ind^−1^)	*n*
Brachycentridae	Trichoptera	Ho	0.35–10.56 (2.96)	24
*Hydropsyche* sp.	Trichoptera	Ho	0.30–32.52 (7.38)	63
Lepidostomatidae	Trichoptera	Ho	3.50–9.20 (4.81)	7
Limnephilidae	Trichoptera	Ho	2.10–33.64 (4.42)	10
*Rhyacophila* sp.	Trichoptera	Ho	1.29–29.81 (11.98)	6
Simuliidae	Diptera	Ho	0.07–3.59 (0.89)	66
*Baetis* sp.	Ephemeroptera	He	0.13–9.18 (1.33)	110
*Ecdyonurus* sp.	Ephemeroptera	He	0.24–25.49 (1.96)	11
*Epeorus* sp.	Ephemeroptera	He	0.44–59.17 (7.71)	91
*Rhithrogena* sp.	Ephemeroptera	He	3.06–21.62 (5.90)	16
*Dinocras cephalotes*	Plecoptera	He	1.72–221.76 (23.12)	138
*Perla marginata*	Plecoptera	He	0.59–150.85 (16.86)	97

Metamorphosis mode: (Ho) holometabolous, (He) hemimetabolous.

Body mass: Range of animal body mass; medians are shown in parenthesis.

*n*: Number of samples for nucleic acid measurements in this study.

## Results

**Figure 1:**
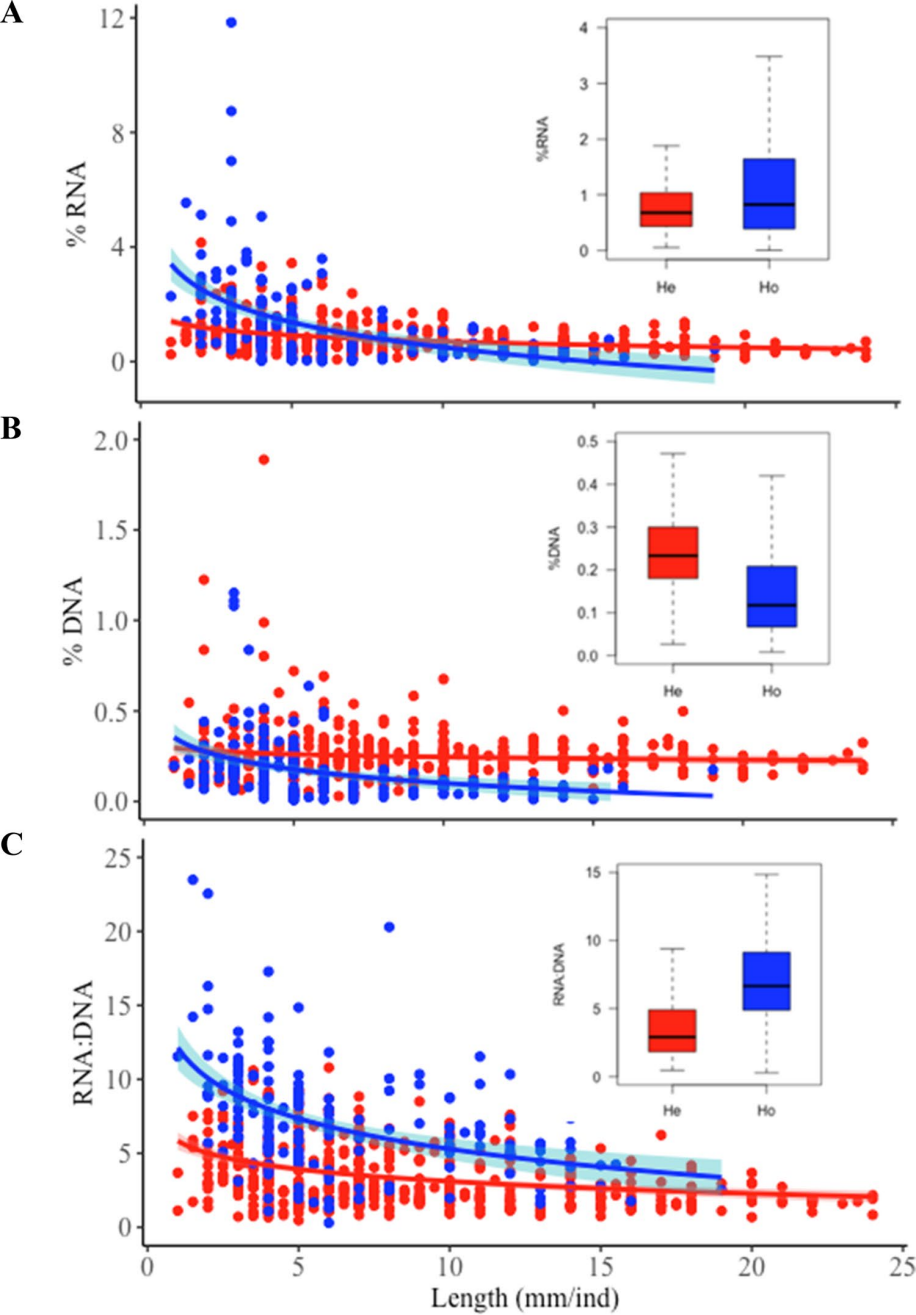
Relationships between body length and nucleic acids. All fits shown were significant and model parameters and statistics for (**A**) RNA, (**B**) DNA and (**C**) RNA:DNA ratio are shown in Table 2. Lines indicate Generalized Linear Model fit and shadows indicate 95% confidence intervals for each fit. Inset box plots display variation in the centerline representing median values, boxes extending to the first and third quartiles, and whiskers to the 1.5 × the interquartile range.

**Figure 2:**
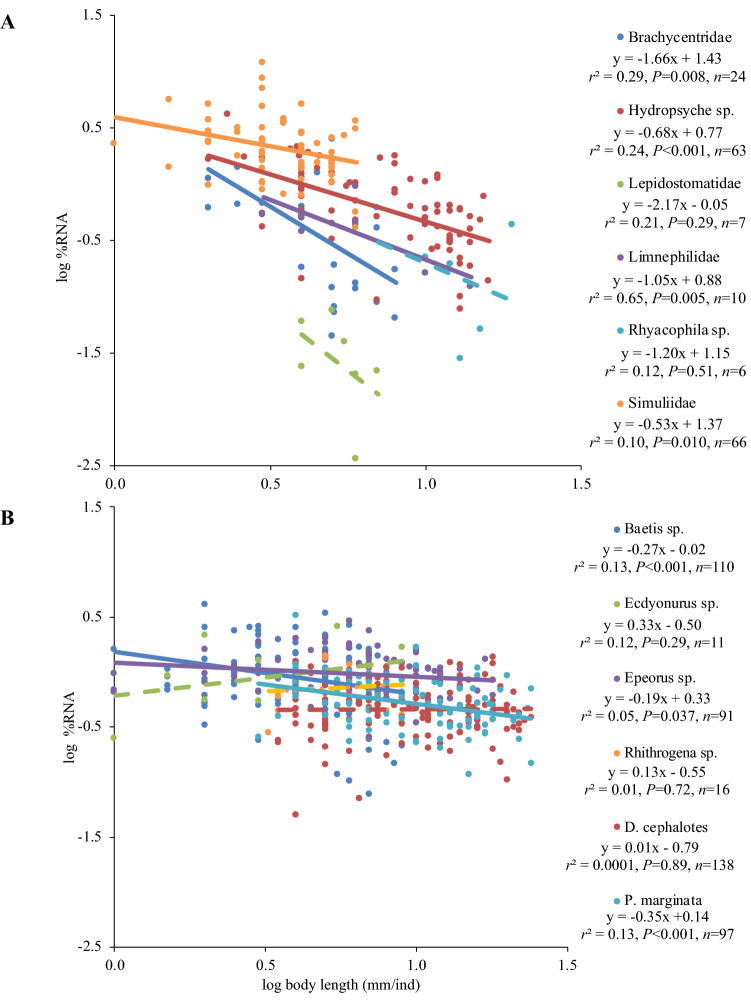
Relationship between body length and %RNA for (**A**) holometabolous and (**B**) hemimetabolous taxa in this study. Equations and regression parameters for linear regressions are included to the side of the figures. Solid lines indicate significant fits, and dashed lines nonsignificant fits.

Our results showed that RNA content was not significantly different between holometabolans and hemimetabolans (median values: 0.82 vs. 0.68, respectively; inset in Fig. 1A and Table 2). We found negative correlations between body length and RNA in four out of six holometabolans, and in three out of six hemimetabolans (Fig. 2). However, negative slopes and the percentage of explained variance were consistently higher in regressions referring to holometabolans. As hypothesised, lower DNA was found in holometabolans relative to hemimetabolans (median values: 0.12 vs. 0.23; inset in Fig. 1B and Table 2). The reported different NA allocation between RNA and DNA was reflected in > twofold higher RNA:DNA ratios in holometabolans (median values: 6.66 vs. 2.91; inset in Fig. 1C), although differences were not statistically significant (*P* < 0.001, Table 2). Also as predicted, body length × metamorphosis mode significantly affected NAs, resulting in pronounced differences in RNA and RNA:DNA ratio between small-sized holometabolan and hemimetabolan insects that diminished as animals grew larger (Fig. 1 and Table 2).

**Table 2:** Generalized linear mixed model results of body length, metamorphosis mode and the interaction between body length and metamorphosis mode as predictors of nucleic acid content (%RNA, %DNA and RNA:DNA) of aquatic macroinvertebrates with taxa nested within order set as random variables.

Response variable	Predictors	Estimated coefficient	Standard error	*t*-value	*P*-value	ΔDIC
%RNA	Body length	0.443	0.111	4.012	** < 0.001**	− 2471.89
	Metamorphosis mode	31.804	23.396	1.359	0.174	
	Body length × Metamorphosis mode	0.777	0.232	3.354	** < 0.001**	
%DNA	Body length	0.205	0.038	5.373	** < 0.001**	− 2088.22
	Metamorphosis mode	21.71	9.627	2.255	**0.024**	
	Body length × Metamorphosis mode	0.0857	0.212	0.403	0.687	
RNA:DNA	Length	0.024	0.034	0.708	0.479	− 1901.03
	Metamorphosis mode	− 3.437	2.220	− 1.548	0.122	
	Body length × Metamorphosis mode	0.258	0.049	5.228	** < 0.001**	

ΔDIC: Difference between gamma model deviance information criteria minus DIC of the binomial model.

Significant results (*P* < 0.05) are indicated in bold.

**Figure 3:**
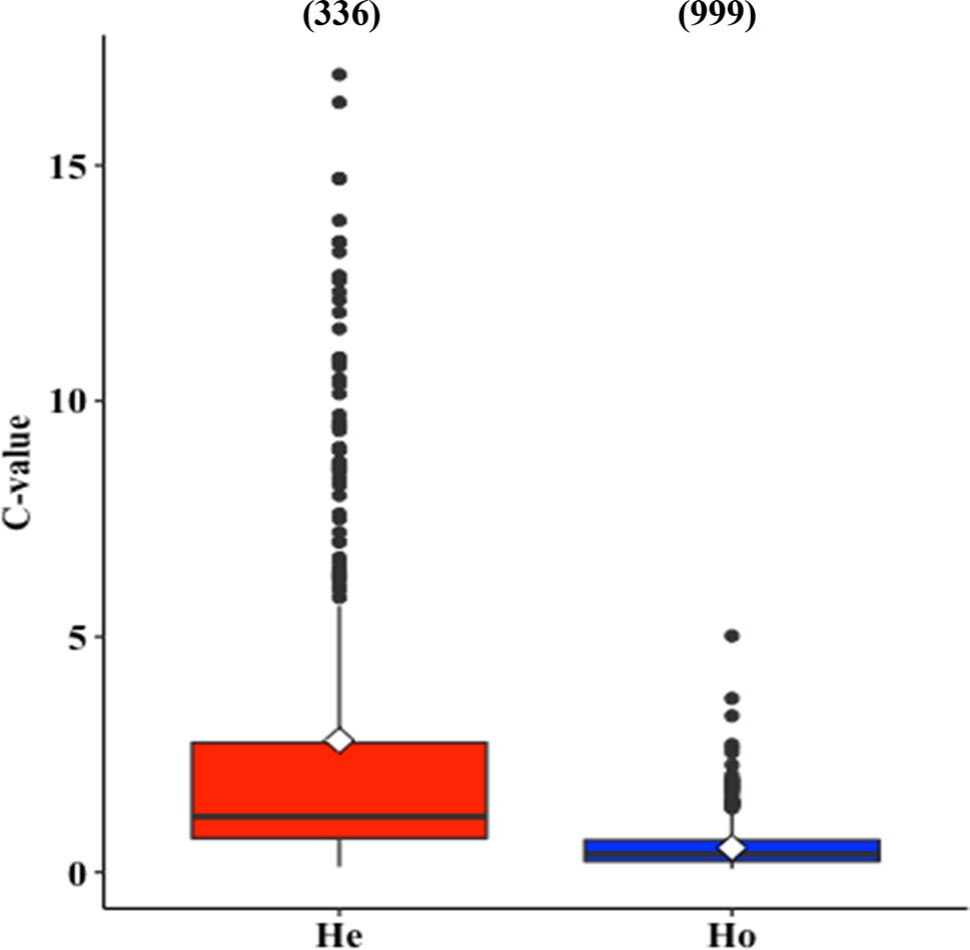
Genome size for insects grouped into hemimetabolous and holometabolous. The C-value (quantity of nuclear haploid DNA) is from a published dataset source (Gregory, 2020; www.genomesize.com). The box plot indicates the variation in the centerline representing median values; boxes extend to the first and third quartiles, whiskers extend to the 1.5 × the interquartile range, and points represent outliers. Diamonds represent group means of 2.79 for hemimetabolans (He) and 0.52 for holometabolans (Ho). Numbers in brackets represent number of observations for each group.

To further assess whether differences in DNA content between metamorphosis modes were representative of pterygote insects, we compared C-values between holo- versus hemimetabolans using the freely-available Animal Genome Size Database^16^. The differences between groups proved highly significant (*P* < 0.001, Table 3), with mean C-values over five times higher in hemimetabolans than in holometabolans (Fig. 3).

**Table 3:** Generalized linear mixed effects model results of metamorphosis mode as a predictor of genome size (C-value) of aquatic macroinvertebrates with order set as a random variable.

Response variable	Predictors	Estimated coefficient	Standard error	*t*-value	*P*-value	ΔDIC
C-value	Metamorphosis mode	15.468	6.829	2.265	**0.024**	− 6843.8

ΔDIC: Difference between gamma model deviance information criteria minus DIC of the binomial model.

Significant results (*P* < 0.05) are indicated in bold.

## Discussion

To our knowledge, this works represents an intensive dataset that for the first time illustrates the pronounced ontogenetic differences in NAs between juvenile insects representing direct and indirect metamorphosis modes. The results support our initial hypotheses of (1) lower DNA content in holometabolans relative to hemimetabolans, and (2) significant body length × metamorphosis mode effects on RNA and RNA:DNA ratio as an indication of pronounced variations in nucleic acid allocation throughout the ontogeny between metamorphosis modes.

**Figure 4:**
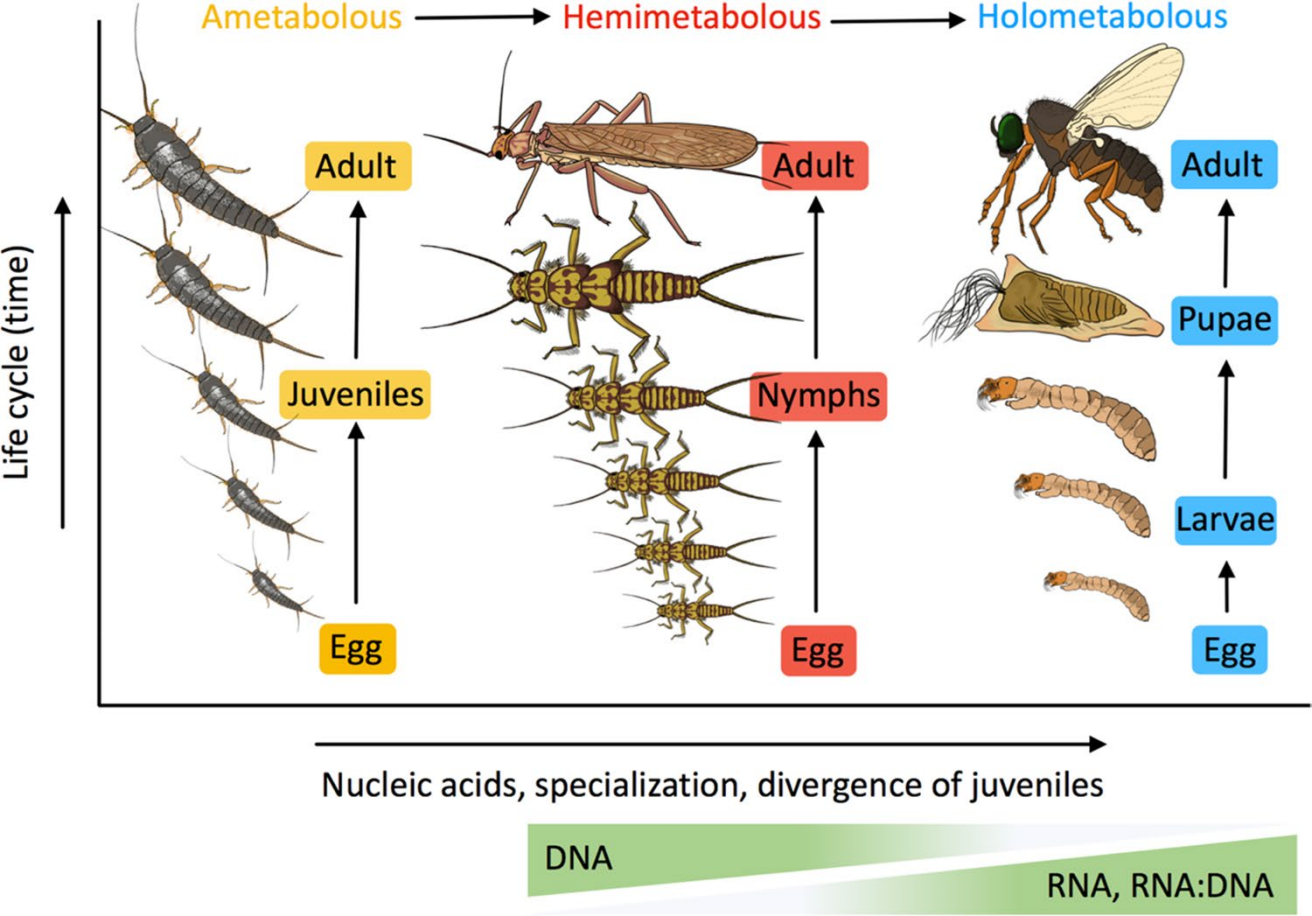
Scheme illustrating the changes in nucleic acid composition in the evolution of insect postembryonic development. Amber represents the ancestral ametabolous mode, red the hemimetabolous mode characterized by nymphs with numerous instars, and blue the holometabolous mode characterized by larvae with fewer moult events before the pupa stage. No ametabolous species were analysed for nucleic acids in this study. The image is credit by I. Peralta-Maraver under CC BY open access copyright, and is based on Belles^3^.

The striking ontogenetic differences found in NA allocation in this study coincide with the divergent evolutionary dynamics in the juvenile development of insects showing contrasting life histories (Fig. 4). In general, hemimetabolous nymphs with lower RNA demands for fast growth have longer life cycles and more numerous moults compared to holometabolans^3^. It has been suggested that the main adaptive benefit of indirect metamorphosis of holometabolans is the decoupling between growth and differentiation^17^. In this process, holometabolous larvae with high RNA undergo fewer moults while gaining and accumulating the nutrients and energy for maximum growth in preparation for pupation. Although growth rate is among the most important life-history traits presumably driving evolutionary fitness^4^, rapid growth might be costly. For example, the lower metabolic cost of growth (i.e. the amount of energy required to synthesise a unit of biomass) in holometabolans can give rise to cells vulnerable to stressors^18^. Our findings, however, suggest a fundamental trade-off between growth and reduced genome size. We found that holometabolans exhibited lower DNA content. This is consistent with their lower genome sizes (C-values) relative to hemimetabolans in the comparison between these two groups using the insect-genome dataset by Gregory^16^, which comprises both aquatic and terrestrial insects (Fig. 3). These findings not only corroborate earlier evidence that the metamorphosis mode could be a primary determinant of the genome-size disparity between holo- and hemimetabolous insects^19,20^, but also support the hypothesis that reallocation of elements (P and N) from DNA to RNA under selection for rapid growth can lead to genome streamlining in eukaryotes^21^.

The observation of substantial ontogenetic differences in NA allocation between RNA and DNA not only provides a mechanistic basis for understanding the eco-evolutionary trade-offs driving metamorphosis diversity, but has far-reaching implications across broad spheres of the ecosystem. Because RNA is directly linked to body-mass production, increased expression of rDNA to produce RNA is directly coupled to secondary production, a process at the foundation of food webs^7,22^. In addition, differences in the allometric patterns of NA allocation between metamorphosis modes might provide a valuable perspective to examine how mixed communities might respond when confronted to nutrient constraints. We might expect early stages of holometabolans with higher demands for P-rich RNA to be particularly sensitive to food quality, as it has been previously shown for copepods^23^.

In the nineteenth century, Lubbock^24^ sought a scientific explanation for why insects undergo metamorphosis, observing that natural selection might operate in juvenile stages of insects with different immature and life-cycle types. Later, in-depth biomolecular information has established a more consistent relation between biochemical composition and evolutionary fitness. That is, differences in nucleic allocation between RNA and DNA vary ontogenetically but, taken together, may reflect fundamental evolutionary pressure towards rapid growth in holometabolans at the expense of diminished genome sizes relative to hemimetabolans. Given the pivotal role that aquatic insects play in freshwater as well as terrestrial food webs, the evolutionary trade-off between RNA and DNA allocation is essential as it influences key life-history traits such as growth rate. In the challenge to establish generalities underlying living systems, the interrelation of body biochemical composition and life-history traits holds considerable promise. According to our results, such an approach provides a suitable mechanistic basis for understanding the ecological fitness and the eco-evolutionary trade-offs driving the success in insects of different metamorphosis modes facing current accelerated environmental changes.

## Methods

### Sample collection

Samples of insects for the analyses of NAs were collected in four basins of Sierra Nevada National Park, southern Spain (Supplementary Fig. S1). Sample sites covered different spatial and temporal scales of investigation: three sampling stations across an elevational gradient and two sampling periods in spring and autumn of 2015. Given that all samples were collected from similar environments, the effect of abiotic conditions was not considered crucial for testing NAs in insects. Samples of aquatic insects were collected using a kick sampler (250 µm mesh size) by removing the substrate from at least 20 sample units (total area of 2.5 m^2^) taken on each station and date and distributed randomly in proportion to the occurrence of major stream habitats (i.e. rapid and slow flow, gravel, sand, zones proximal to and distant from shore and vegetated areas). All samples from each station were pooled and individuals representing six hemimetabolous and six holometabolous taxa sorted specifically for the analysis of nucleic acids (Table 1). We refer to taxa as a generic term to designate a group of one or more populations of organisms that were identified to the lowest taxonomic level possible by eye. Thus, most taxa were identified to the species (*Dinocras cephalotes,* and *Perla marginata*) or genus level (*Baetis* sp., *Ecdyonurus* sp., *Epeorus* sp., *Rhithrogena* sp., *Hydropsyche* sp., and *Rhyacophila* sp.), except for Lepidostomatidae, Limnephilidae, Brachycentridae, and Simuliidae﻿ that were identified to the family level. Although taxonomic resolution in the identification varied, taxa identified at the species and genus level represented the majority of the samples in this study. In addition, morphologically similar animals were selected for all supraspecific taxa in order to represent similar morphospecies for each taxon. When possible, up to 20 individuals per taxa that covered the full size spectrum available for each taxon were sorted into 10-mL vials containing RNAlater (Ambion Inc., Austin, Texas, USA), and transported inside a cooler to the laboratory. There, all insect samples were stored at − 80 °C until prepared for analysis. Before processing the insects, we measured body length to the nearest half millimetre under a stereoscopic microscope and verified the insect’s identity. In total, 639 individuals of 12 different taxa (six hemimetabolans and six holometabolans) were measured and analysed for NA content.

### Nucleic acid analysis

NA analyses largely followed the methods by Wagner et al.^13^ with a number of recommendations by Gorokhova & Kyle^25^ and Bullejos et al.^26^. Analyses were carried out on insect legs and/or heads except for Simuliidae, where entire individuals were analysed. Preliminary analyses using legs and heads for a given individual showed that the coefficient of variation in RNA and DNA content rarely exceeded 5% (Supplementary Table S1). For the calculation of dry weight of insects where legs (one to three) were analysed, the opposite legs and the remaining body parts were separately weighed to estimate total body dry mass (total body weight = legs dry weight * 2 + remaining body parts dry weight). For the estimation of dry weight of insects where heads or the entire body were analysed, body length–weight relationships were specifically developed for each taxon in this study (Supplementary Table S2). Dry-weight was estimated by drying samples to constant weight in preweighed aluminium capsules and reweighing them with a Mettler UMT2 microbalance (± 0.1 µg; Mettler Toledo, Im Langacher, Switzerland).

NAs were measured using a microplate fluorimetric high-range assay Ribo-Green assay (Initrogen, Carlsbad, California, USA) after N-laurylsarcosine extraction and RNase digestion, as described in Gorokhova & Kyle^25^. We used the following reagents: RiboGreenTM RNA Quantitation Kit (Invitrogen Corporation, Carlsbad, California, USA); RNase DNasefree (working solution 5 mg mL21; Q-biogen, Weston, Massachusetts, USA); N-lauroysarcosine (Sigma-Aldrich, Saint Louis, Missouri, USA); Tris-EDTA buffer (Q-biogene). Fluorescence measurements were performed using a FLUOstar Optima fluorometer (microplate reader, filters: 485 nm for excitation and 520 nm for emission; BMG Labtechnologies, Ortenberg, Germany) and black solid flat-bottom microplates (Greiner Bio-One GmbH, Frickenhausen, Germany). The plate was scanned with a 0.2-s well measurement time, making 10 measurements per well, before and after RNase digestion (30 min under dark conditions at 37 °C). Fluorescence measurements were converted into RNA and DNA concentrations (pg) by using standard curves for RNA (16S and 23S from *Escherichia coli*, component C of the RiboGreen Kit) and DNA (calf thymus; Sigma-Aldrich), and expressed as a percentage of body dry mass (%RNA and %DNA).

### Animal genome size database

To test the generality of our hypothesis that DNA size varied between insect metamorphosis modes across taxa and environments (terrestrial and aquatic), we incorporated the Animal Genome Size Database by Gregory^16^ in our analysis. The dataset covers a variety of insect groups (including 140 families and 20 orders of hemimetabolous and holometabolous insects) with a representation of most functional feeding groups, life cycles, and trait-based morphologies, comprising a total of 336 hemimetabolous and 999 holometabolous insect records.

### Statistical analysis

Testing for differences in NAs between metamorphosis modes, we found that data were not normally distributed (Shapiro–Wilk’s *W* test) and could not be transformed to fit a normal distribution, so differences in NAs were tested using generalized linear mixed effects models (GLMM). Models included body length and metamorphosis mode as fixed factors, and insect taxa nested within order as random factors to account for variability within taxa subgroups. The significance of the interaction between body length and metamorphosis mode was used to test whether NA allocation to RNA differed during the ontogenetic development of animals. To examine whether insect genome size (C-value) varied between holo- and hemimetabolans using Gregory’s genome size dataset, a GLMM was also used with metamorphosis mode set as a predictor and taxa nested within order as a random variable. Before performing the models, the data were standardized (Deconstand function in R) to provide meaningful estimates of main effects in models with interaction terms^27^ and the best GLMM was selected according to deviance information criteria^28^. GLMM analyses were conducted using the ‘*glmer*’ function in the package ‘*lme4*’^29^. Finally, because NA data for taxon subsets were normally distributed after a log-transformation, linear-regression models were used to test the relationship between RNA and body length for each taxon. All statistical analyses were made in R^30^.

## Supplementary Information

Supplementary Information.

## Data Availability

The datasets generated during and/or analysed during the current study are available from the corresponding author on reasonable request.

## References

[1] Zhang ZQ (2011). An outline of higher-level classification and survey of taxonomic richness. Zootaxa.

[2] Nation, J. L., Sr. *Insect Physiology and Biochemistry,* 3rd edn (CRC Press, 2016).

[3] Belles X (2020). Insect Metamorphosis: From Natural History to Regulation of Development and Evolution.

[4] Sterner, R. W., & Elser, J. J. *Ecological Stoichiometry* (Princeton University Press, 2002).

[5] Villar-Argaiz M, Medina-Sánchez JM, Carrillo P (2002). Linking life history strategies and ontogeny in crustacean zooplankton: implications for homeostasis. Ecology.

[6] Main TM, Dobberfuhl DR, Elser JJ (1997). N: P stoichiometry and ontogeny of crustacean zooplankton: a test of the growth rate hypothesis. Limnol. Oceanogr..

[7] Elser JJ (2003). Growth rate–stoichiometry couplings in diverse biota. Ecol. Lett..

[8] Bullejos FJ, Carrillo P, Gorokhov E, Medina-Sánchez JM, Villar-Argaiz M (2014). Nucleic acid content in crustacean zooplankton: bridging metabolic and stoichiometric predictions. PLoS ONE.

[9] Villar-Argaiz M, López-Rodríguez MJ, Tierno de Figueroa JM (2020). Body P content increases over ontogeny in hemimetabolous macroinvertebrates in a Mediterranean high mountain stream. Aquat. Ecol..

[10] Cole BJ (1980). Growth ratios in holometabolous and hemimetabolous insects. Ann. Entomol. Soc. Am..

[11] Traganos F, Darzynkiewicz Z, Melamed MR (1982). The ratio of RNA to total nucleic acid content as a quantitative measure of unbalanced cell growth. Cytometry.

[12] Bergeron JP (1997). Nucleic acids in icthyoplankton ecology: a review, with emphasis on recent advances for new perspectives. J. Fish Biol..

[13] Wagner MM, Durbin E, Buckley L (1998). RNA:DNA ratios as indicators of nutritional condition in the copepod *Calanus finmarchicus*. Mar. Ecol. Prog. Ser..

[14] Lynch, M. *The Origins of Genome Architecture* (Sinauer Associates, 2007).

[15] Hessen DO, Ventura M, Elser JJ (2008). Do phosphorus requirements for RNA limit genome size in crustacean zooplankton?. Genome.

[16] Gregory, T. R. *Animal Genome Size Database* (World wide web electronic publication, accessed 27 November, 2020). http://www.genomesize.com.

[17] Rolff J, Johnston PR, Reynolds S (2019). Complete metamorphosis of insects. Philos. Trans. R. Soc. Lond. B Biol. Sci..

[18] Ferral N (2020). The extremely low energy cost of biosynthesis in holometabolous insect larvae. J. Insect Physiol..

[19] Gregory TR (2005). The Evolution of the Genome.

[20] Alfsnes K, Leinaas HP, Hessen DO (2017). Genome size in arthropods; different roles of phylogeny, habitat and life history in insects and crustaceans. Ecol. Evol..

[21] Hessen DO, Punidan D, Jeyasingh PD, Neiman M, Weider LJ (2009). Genome streamlining and the elemental cost of growth. Trends Ecol. Evol..

[22] Elser JJ (2006). Biological stoichiometry: a chemical bridge between ecosystem ecology and evolutionary biology. Am. Nat..

[23] Villar-Argaiz M, Sterner RW (2002). Life history bottlenecks in *Diaptomus clavipes* induced by phosphorus-limited algae. Limnol. Oceanogr..

[24] Lubbock J (1873). On the origin and metamorphoses of insects. Nature.

[25] Gorokhova E, Kyle M (2002). Analysis of nucleic acids in *Daphnia*: development of methods and ontogenetic variations in RNA-DNA content. J. Plankton Res..

[26] Bullejos FJ, Carrillo P, Gorokhova E, Medina-Sánchez JM, Balseiro EG, Villar-Argaiz M (2014). Shifts in food quality for herbivorous consumer growth: multiple golden means in the life history. Ecology.

[27] Murray, D. L. *et al.**Population Ecology in Practice* (Wiley, 2020).

[28] Bolker BM (2009). Generalized linear mixed models: a practical guide for ecology and evolution. Trends Ecol. Evol..

[29] Bates D, Mächler M, Bolker B, Walker S (2015). Fitting linear mixed-effects models using lme4. J. Stat. Softw..

[30] R Development Core Team R: *A Language and Environment for Statistical Computing* (R Foundation for Statistical Computing, Austria, 2016).

