# Supplementary material for: Divergent nucleic acid allocation in juvenile insects of different metamorphosis modes
Source: Sci Rep. 2021 May 13;11:10313. doi: 10.1038/s41598-021-89736-w (PMC8119467; doi:10.1038/s41598-021-89736-w)
Supplement: Supplementary file 1 — Supplementary Information. [file 41598_2021_89736_MOESM1_ESM.docx]

**Supplementary information for**

**Divergent nucleic acid allocation in juvenile insects of different metamorphosis modes**

Manuel Villar-Argaiz^1*^, Manuel J. López-Rodríguez^1^, J. Manuel Tierno de Figueroa^2^

^1^Departamento de Ecología, Facultad de Ciencias, Universidad de Granada, 18071 Granada, Spain

mvillar@ugr.es, manujlr@ugr.es

^2^Departamento de Zoología, Facultad de Ciencias, Universidad de Granada, 18071 Granada, Spain

jmtdef@ugr.es

*Corresponding author

**Supplementary Figure S1, Supplementary Table S1, Supplementary Table S2**

**Supplementary Figure S1.** Map of the study stations in the four basins of Sierra Nevada in southern Spain (G=Genil, D=Dílar, P=Poqueira, T=Trevélez). Shaded grey area marks the limits of Sierra Nevada Natural Park. Numbers after the basin code represent sampling stations (green pushpins) which were distributed across an altitudinal gradient, station 1 being a high-mountain site above the tree line (>2500m), station 2 an intermediate site with riparian vegetation and a primary forest canopy (~1500 m), and station 3 (between 1000-1300m) a lower site downstream of population centres (red areas). Map was made in QGIS (version 3.18; https://qgis.org/es/site/).

**
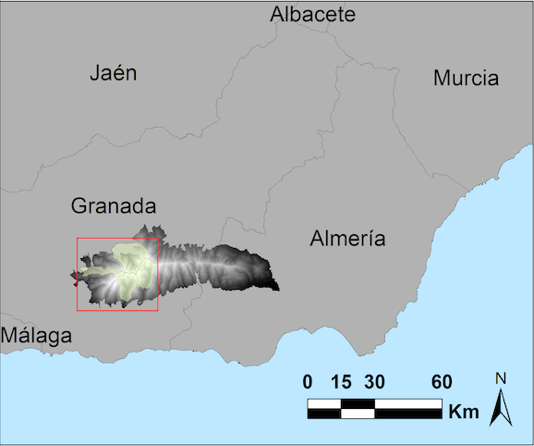

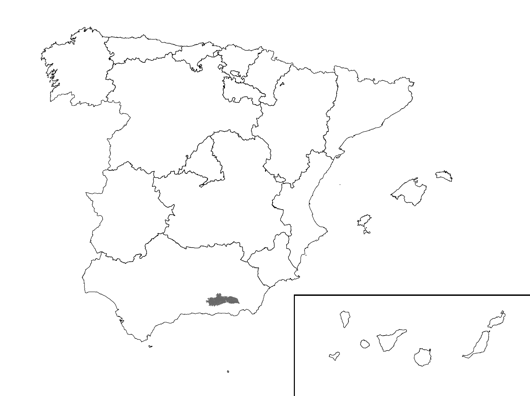

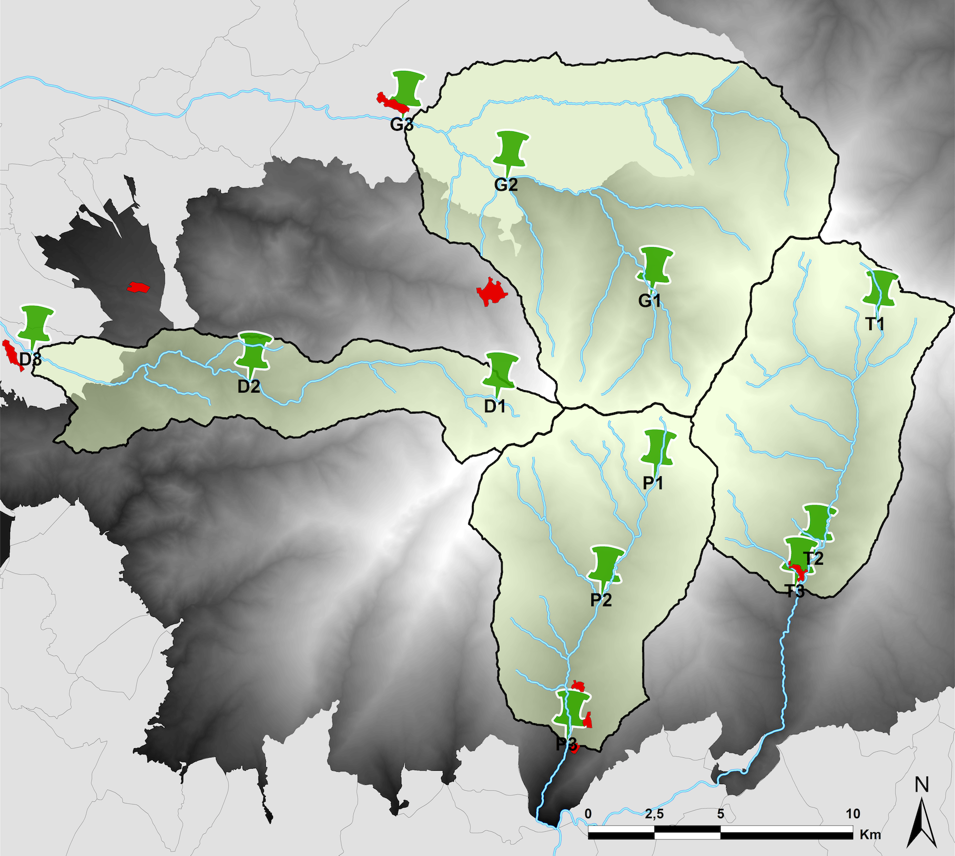
**

**Supplementary Table S1.** Coefficients of variation of nucleic acid analyses using different body parts of single insect measurements.

| **Taxa** | **Sample #** | **Body parts analysed** | **DNA**  **(%CV)** | **RNA**  **(%CV)** | **RNA:DNA (%CV)** |
| --- | --- | --- | --- | --- | --- |
| *Rhithrogena* sp. | 1 | 3 legs | 0.110 (4.0) | 0.440 (0.7) | 4.009 (3.2) |
|  |  | head | 0.116 | 0.444 | 3.829 |
|  | 2 | 3 legs | 0.130 (6.8) | 0.578 (2.7) | 4.451 (4.1) |
|  |  | head | 0.143 | 0.601 | 4.203 |
|  | 3 | 3 right legs | 0.263 (0.6) | 1.159 (0.7) | 4.410 (0.1) |
|  |  | 3 left legs | 0.265 | 1.170 | 4.415 |
| *Baetis* sp. | 4 | 3 legs | 0.194 (9.3) | 1.359 (2.2) | 7.015 (7.1) |
|  |  | head | 0.221 | 1.402 | 6.344 |
|  | 5 | 3 legs | 0.183 (3.2) | 1.612 (3.3) | 8.833 (0.1) |
|  |  | head | 0.191 | 1.689 | 8.843 |
|  | 6 | 3 right legs | 0.208 (2.0) | 1.202 (2.4) | 5.782 (0.3) |
|  |  | 3 left legs | 0.214 | 1.243 | 5.808 |
| *Perla marginata* | 7 | 1 right leg | 0.311 (4.1) | 0.515 (0.4) | 1.653 (4.5) |
|  |  | 1 left leg | 0.330 | 0.512 | 1.484 |
| *Hydropsyche* sp. | 8 | 2 right legs | 0.118 (8.8) | 0.580 (7.7) | 4.925 (1.1) |
|  |  | 2 left legs | 0.133 | 0.647 | 4.846 |

%CV = coefficient of variation (standard deviation of nucleic acid measurements / overall mean of nucleic acid measurements ∗ 100)

**Supplementary Table S2.** Dry weight-length relationships developed specifically for the aquatic insect taxa analysed in this study. DW= dry weight (mg ind^-1^); BL=body length (mm ind^-1^). Model: log DW= *a* * log BL + log *b*.

| **Taxa** | **Order** | **Developmental mode** | ***a*** | ***b*** | ***n*** | ***r*^2^** |
| --- | --- | --- | --- | --- | --- | --- |
| Brachycentridae | Trichoptera | Ho | 0.109 | 1.995 | 24 | 0.63 |
| *Hydropsyche* sp. | Trichoptera | Ho | 0.067 | 2.096 | 63 | 0.88 |
| Lepidostomatidae | Trichoptera | Ho | 0.264 | 1.828 | 7 | 0.90 |
| Limnephilidae | Trichoptera | Ho | 0.302 | 1.816 | 10 | 0.96 |
| *Rhyacophila* sp. | Trichoptera | Ho | 0.002 | 3.453 | 6 | 0.94 |
| Simuliidae | Diptera | Ho | 0.079 | 1.742 | 66 | 0.57 |
| *Baetis* sp. | Ephemeroptera | He | 0.098 | 1.902 | 110 | 0.71 |
| *Ecdyonurus* sp. | Ephemeroptera | He | 0.192 | 1.873 | 11 | 0.89 |
| *Epeorus* sp. | Ephemeroptera | He | 0.164 | 1.926 | 91 | 0.92 |
| *Rhithrogena* sp. | Ephemeroptera | He | 0.197 | 2.172 | 16 | 0.95 |
| *Dinocras cephalotes* | Plecoptera | He | 0.108 | 2.344 | 138 | 0.94 |
| *Perla marginata* | Plecoptera | He | 0.043 | 2.552 | 97 | 0.95 |
